# Supplementary material for: Super‐resolved local recruitment of CLDN5 to filtration slits implicates a direct relationship with podocyte foot process effacement
Source: J Cell Mol Med. 2021 Jun 22;25(16):7631–41. doi: 10.1111/jcmm.16519 (PMC8358871; doi:10.1111/jcmm.16519)
Supplement: Supplementary file 1 — Fig S1‐S6 [file JCMM-25-7631-s001.pptx]

## Slide 1
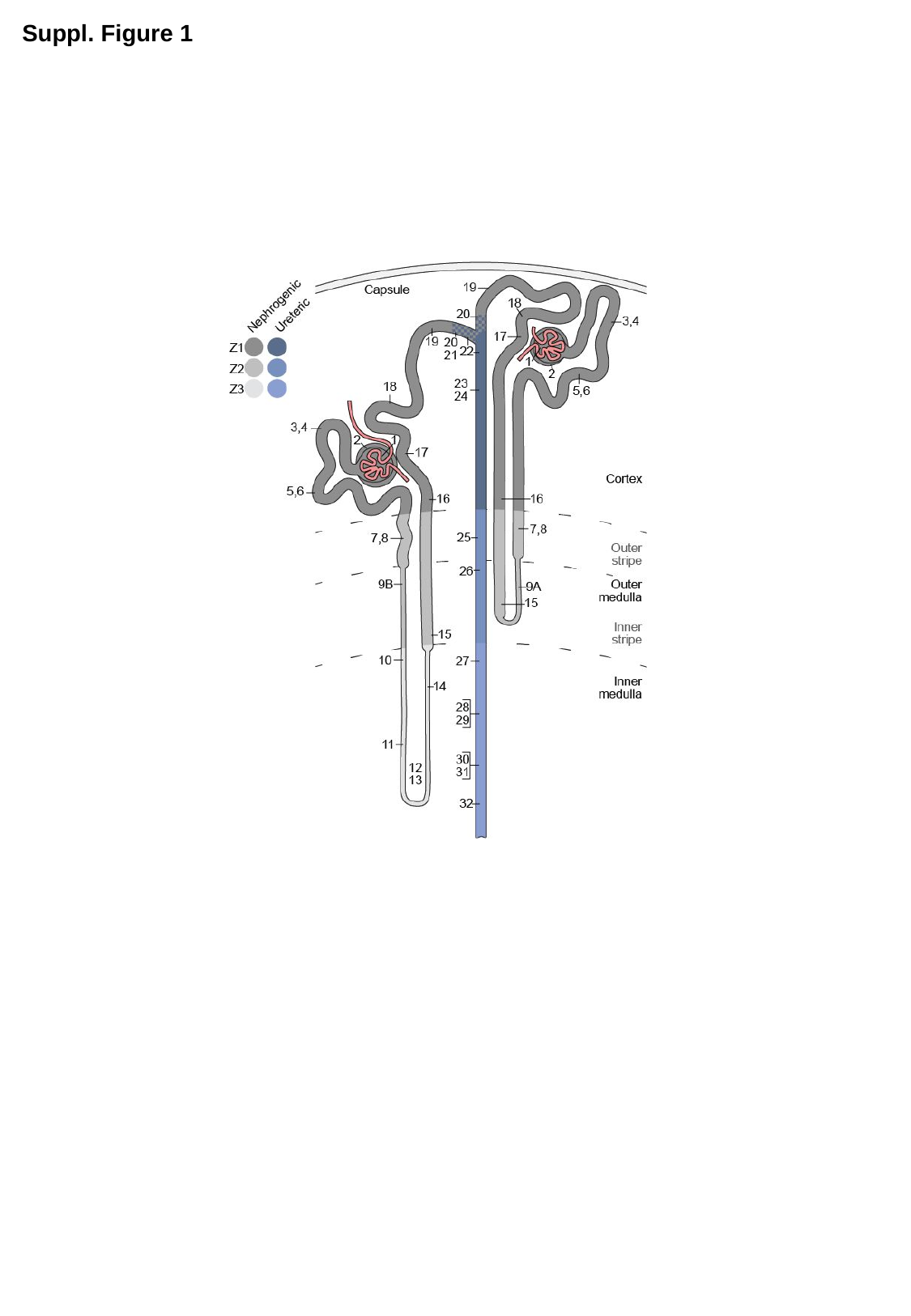

Suppl. Figure 1

## Slide 2
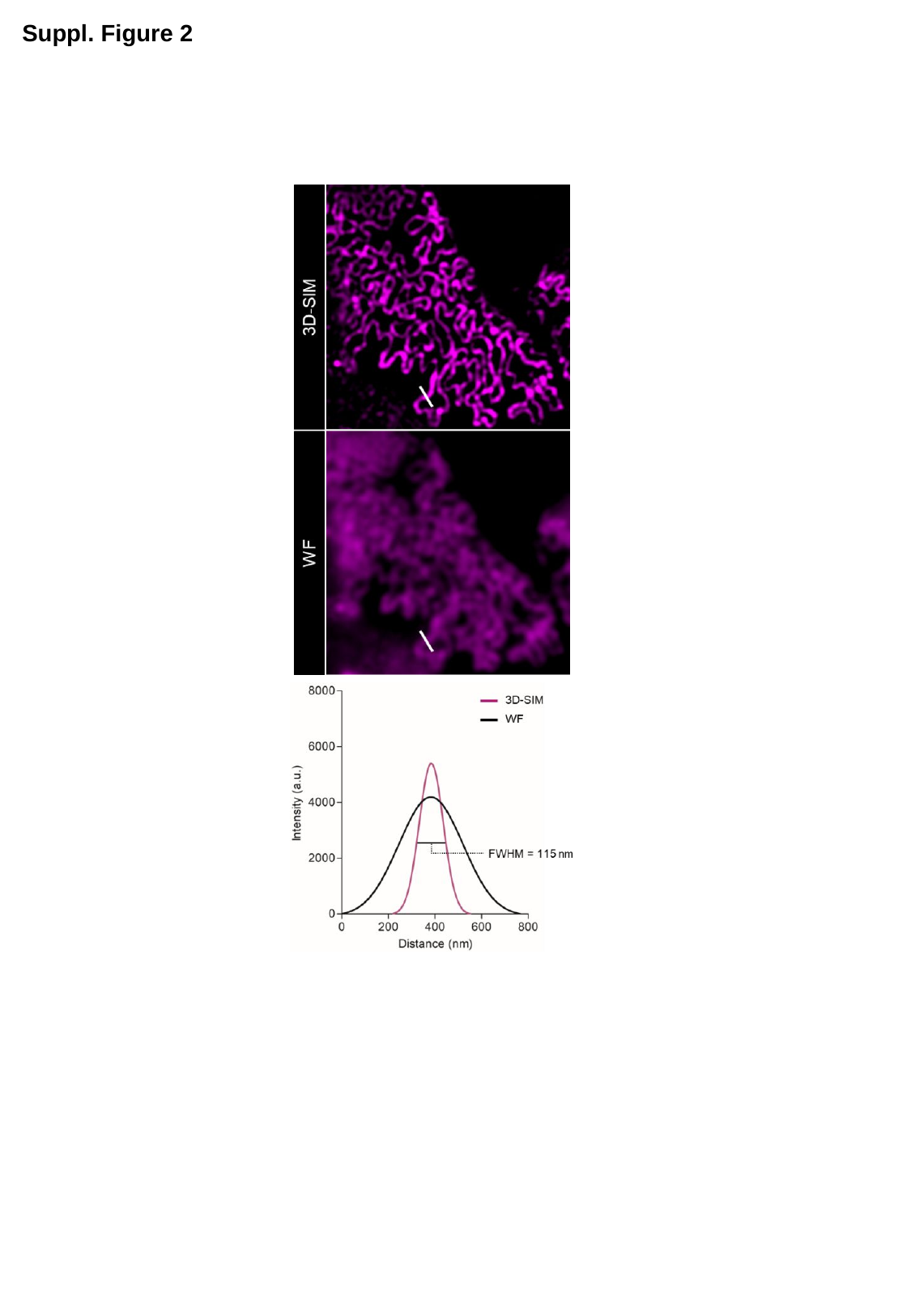

Suppl. Figure 2

## Slide 3
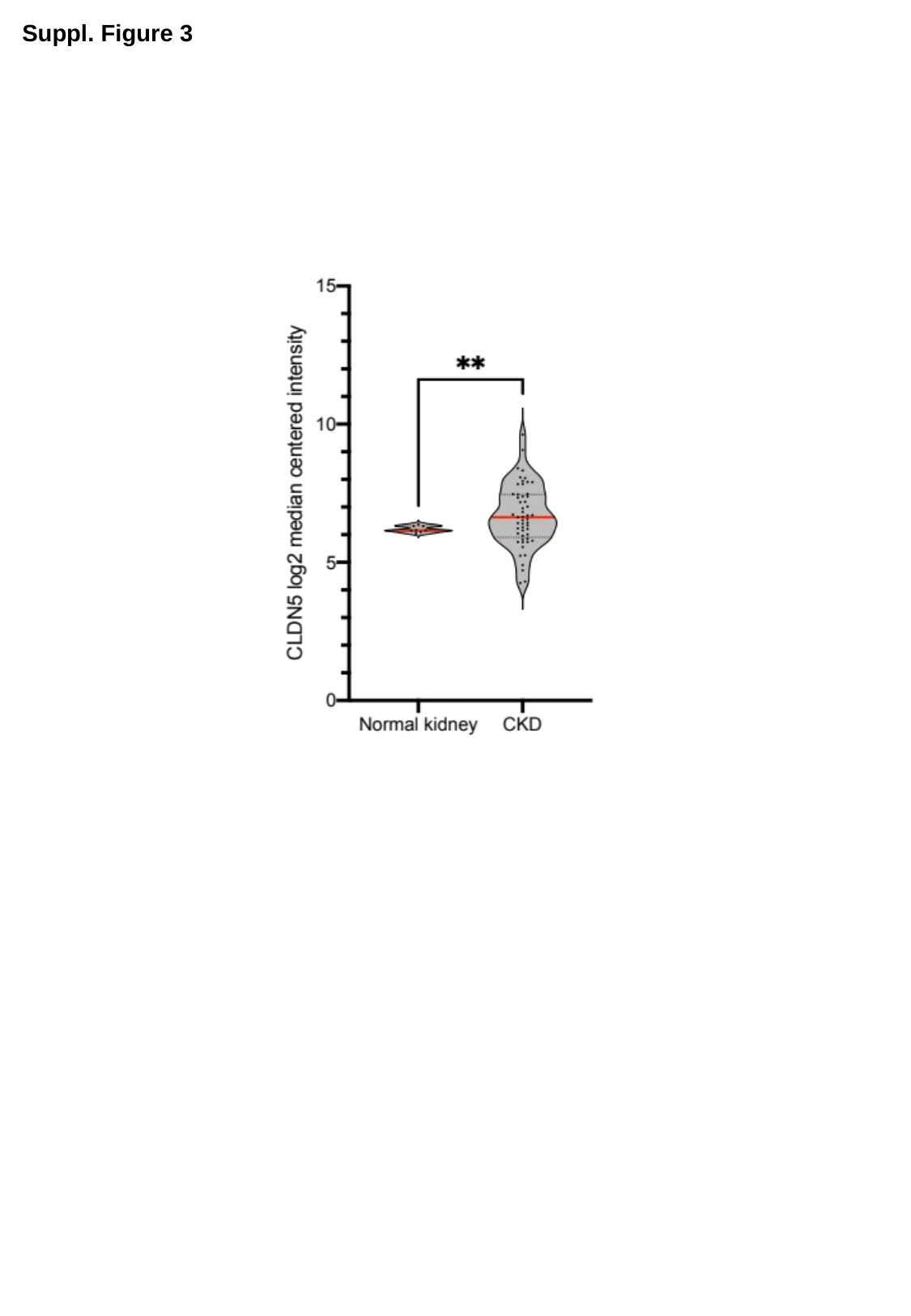

Suppl. Figure 3

## Slide 4
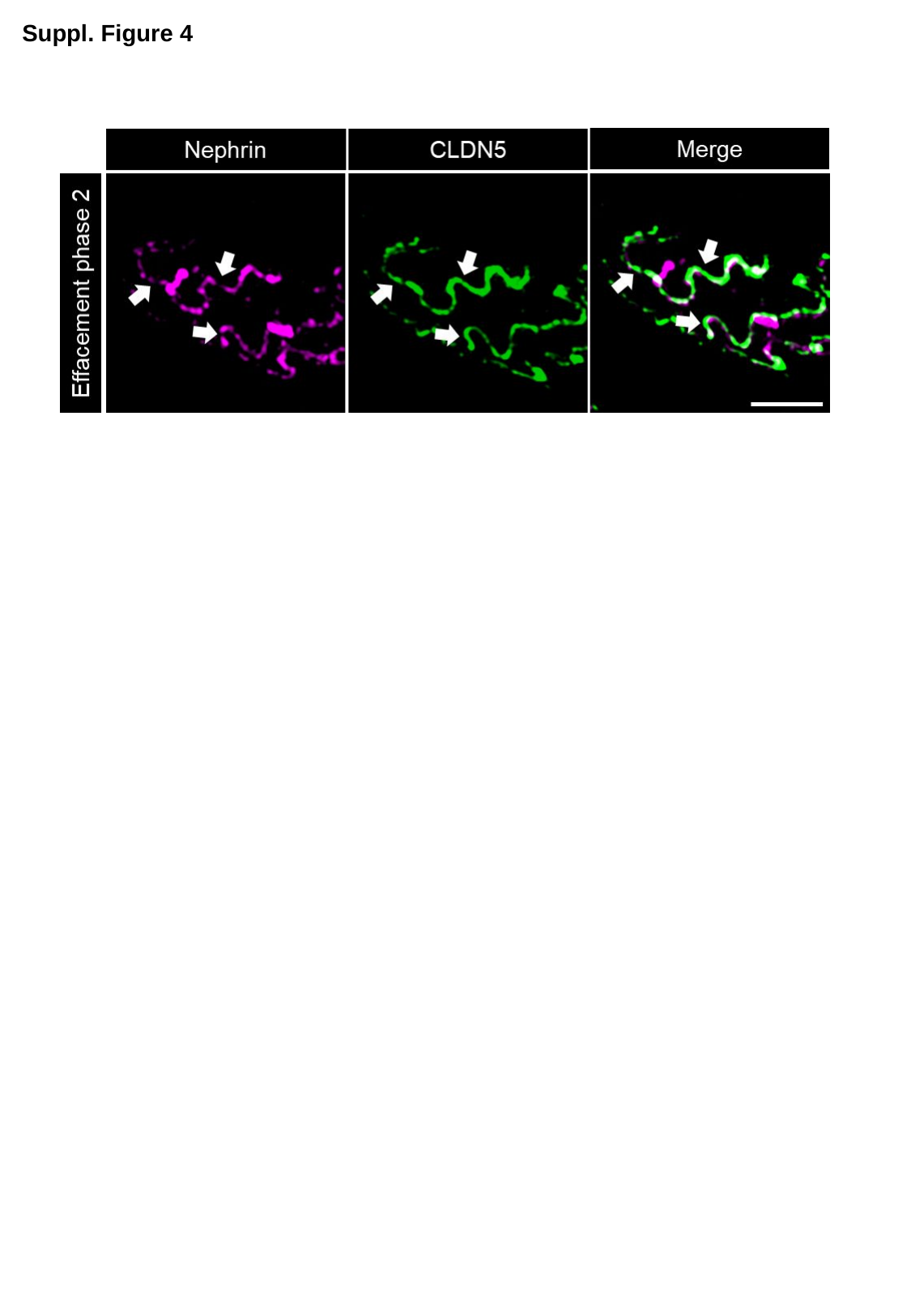

Suppl. Figure 4

## Slide 5
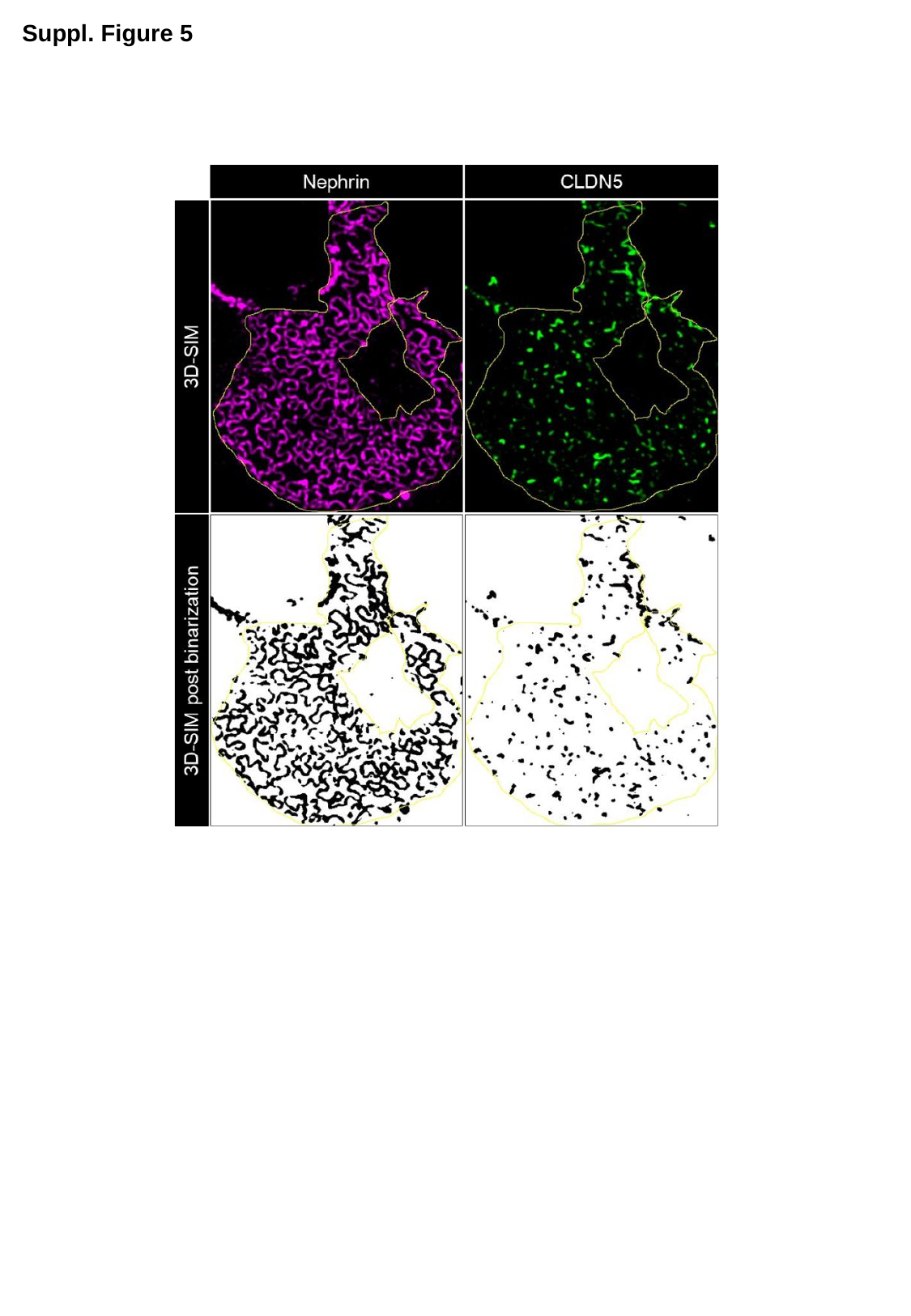

Suppl. Figure 5

## Slide 6
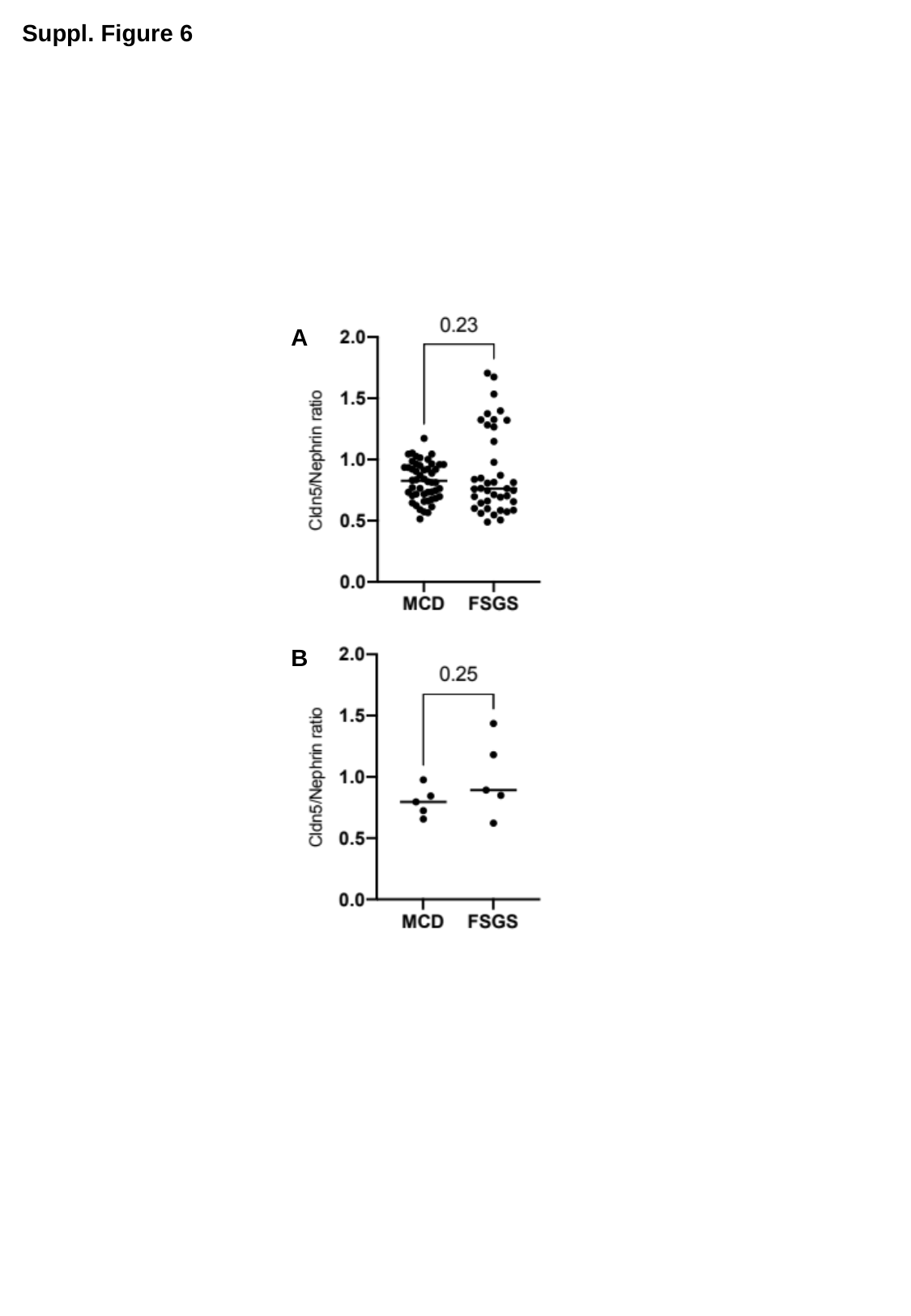

Suppl. Figure 6
A
B
